# The Query

**Published:** 1874-11

**Authors:** 


### THE QUERY.

Sits the sphinx beside me daily,
Whether I be sad or gayly
Live my life. "Attend," she cries—
Looks me through with solemn eyes;
"Thou must answer, answer this:
What the sum of woe and bliss?
What is life?"
In all gladness, through all pain,
Whether peace or passion reign,
Turn my thoughts to things of earth
Or to themes of heavenly birth,
Still I hear that undertone,
Like the ocean's distant moan,
"What is life?"
Once I thought that I might trance her
Into silence by an answer;
Thought that I could find the reason,
I could measure time and season,
I could sound the depths she stirred,
I could compass with a word
What is life!
Youthful, vain, and fond delusion!
Now I turn from light intrusion
On the secret that she keeps
Close within her stony lips,
Which but open to their task,
Ne'er to answer, only ask,
"What is life?"
Hark! she whispers, "Thou shalt die
If thou givest no reply."
Once with shuddering and with pain
Flashed her words through every vein!
Now I wait the parting breath,
When shall answer friendly Death
What is life!

